# Journalism, public health, and COVID-19: some preliminary insights from the Philippines

**DOI:** 10.1177/1329878X20953854

**Published:** 2020-11

**Authors:** Jan Michael Alexandre C Bernadas, Karol Ilagan

**Affiliations:** De La Salle University, Philippines

**Keywords:** COVID-19, critical analysis of media and public health, journalism, Philippines, public health

## Abstract

In this essay, we engage with the call for Extraordinary Issue: Coronavirus, Crisis and Communication. Situated in the Philippines, we reflect on how COVID-19 has made visible the often-overlooked relationship between journalism and public health. In covering the pandemic, journalists struggle with the shrinking space for press freedom and limited access to information as they also grapple with threats to their physical and mental well-being. Digital media enable journalists to report even in quarantine, but new challenges such as the wide circulation of health mis-/disinformation and private information emerge. Moreover, journalists have to contend with broader structural contexts of shutdown not just of a mainstream broadcast but also of community newspapers serving as critical sources of pandemic-related information. Overall, we hope this essay broadens the dialogue among journalists, policymakers, and healthcare professionals to improve the delivery of public health services and advance health reporting.

## Introduction

In this essay, we reflect on how COVID-19 has brought to our attention the often-overlooked relationship between journalism and public health. We draw initial insights from critical analysis of media and public health (Henderson and Hilton, 2018) to suggest that health reporting in the country during the pandemic can be connected to journalistic practices, technological changes, and structural constraints. For journalism to advance public health, it needs to contend with the pandemic and the context into which it is uniquely situated – both of which are moving targets and difficult to predict. In this essay, we pay attention to the Philippines not just because it has one of the highest COVID 19-related cases and deaths in the world but also because the country is at the crossroads of changes in digital media and shrinking space for media freedom, as evidenced by the shutdown of the country’s biggest media network, closing or suspension of community newspapers, and passage of laws that may restrict free speech. In doing so, we hope to broaden dialogue among journalists, policymakers, and healthcare professionals to improve the delivery of public health services as well as advance health reporting.

Similar to other countries, the public health system in the Philippines was unprepared for and overburdened by COVID-19. The first case was reported on January 30 when a Chinese woman reached the country from Wuhan, China, and then a few days later her male companion died of the virus – making it the first recorded death outside of China (Department of Health (DOH), 2020b; Ramzy and May, 2020; World Health Organization (WHO), 2020a). By March 7, the first case of local transmission was confirmed (DOH, 2020a; WHO, 2020a). To date, there are 112,593 confirmed cases, 6,263 new cases, and 2,115 deaths in the country (WHO, 2020b) – making the Philippines as one of the most highly impacted in Southeast Asia and the Western Pacific Region. Equally alarming is the number of doctors, nurses, and other hospital staff who get infected and die of COVID-19 (CNN Philippines, 2020a; McCarthy, 2020). Recently, professional medical and allied medical associations have called for a unified and calibrated response and temporary quarantine of the country’s capital to avoid a total collapse of the healthcare system (Batnag, 2020). Critical but seldom discussed are the challenges of journalism in making sense of the rapid spread and devastating impact of COVID-19 in the Philippines and how the pandemic is also gradually transforming journalism in the country.

Journalism and public health work together to broaden health information sources, facilitate public understanding of health, and mobilize support for or against public health policy (Henderson and Hilton, 2018; Larsson et al., 2003; Vercellesi et al., 2010) and this relationship is magnified during pandemics. The relationship between journalism and public health has mostly been explained based on journalistic roles and news framing. During the 2009 H1N1, for instance, Klemm et al. (2017) found that journalists shifted from ‘watchdogs’ to ‘cooperative’ roles. Holland et al. (2014) further argued that the 2009 H1N1 enabled journalists to be reflexive of their roles especially with conflicts of interest among experts and decision makers. News framing has likewise informed the conversations between journalism and public health. For example, Krishnatray and Gadekar (2014) found that fear and panic dominated the frames used by journalists in their news stories about the 2009 H1N1. In this essay, we hope to engage with ongoing discussion about journalism and public health by reflecting on how health reporting during COVID-19 in the Philippines relates to broader, emergent, and interconnected issues of journalistic practices, technological changes, and structural constraints in the country.

### Reporting from home

COVID-19, along with the ensuing quarantines, poses challenges to existing journalistic practices that typically require fieldwork, but it also encourages journalists in the Philippines to reimagine news production. We observe that access to information has generally been limited because government offices have not been in full operation while virtual press briefings do not allow for a more open discussion between journalists and officials. To illustrate, Ilagan (2020) reported that most routine requests for information have not been processed since March 2020 when government offices were wholly or partly closed due to the ongoing quarantine. The Philippines is among many governments in the world that had to suspend the processing of freedom-of-information (FOI) requests because of the pandemic (McIntosh, 2020). FOI officers working from home could not address requests because they lacked Internet connection, laptop computers, and scanners, including digital copies of files. They also found it difficult to coordinate remotely with record custodians. While some national agencies have been proactive in providing information on COVID-19, the same cannot be said for many local government units. Ilagan (2020) further noted that ‘[un]like frontline agencies at the national level, local governments do not proactively publish data on their websites’. Information about plans to combat the impact of the virus are usually available, but more prodding is needed to find out how these plans are being implemented and funded. Camus (2020) also reported that journalists were prohibited from covering what is happening in hospitals and other high-risk areas. More and more press briefings have thus taken place online, but reporters have found it harder to demand answers because officials and their staff often screen questions. For instance, Camus (2020) wrote that some questions from journalists were ignored while official reports from the government were consistently discussed.

Moreover, we observe that the pandemic has taken a toll on both the physical and mental well-being of journalists. Reported cases of journalists experiencing high levels of stress, undergoing self-quarantines, and at least one news anchor contracting the virus point to the need for broader safety measures at the organizational level of news outlets. The National Union of Journalists of the Philippines (NUJP) lamented the limited mental health support for journalists by saying that ‘there are hardly any readily available and sustained support systems for colleagues experiencing mental health issues’ (Adel, 2020). Safeguarding the physical and psychological well-being of journalists during pandemics or any type of crisis does not rest on individuals alone but should be demanded from news organizations and advocated for by professional associations. Yet some journalists have been able to navigate the consequences of COVID-19 on the profession by reimagining newsgathering, taking advantage of online resources as well as doing collaborations.

First, journalists have been coping with the challenge of limited access to information by interviewing sources through phones and attending webinars with experts to learn more about the pandemic (Tantuco, 2020). Bolledo (2020) said that journalists had to adapt in light of the global health crisis changing media operations. By adapting, he referred to Reuters’ approaches to comprehensive newsgathering, which focus on open-source and non-mainstream techniques such as ‘citizen and collaborative journalism’ and ‘social journalism’. In practice, this set of methods includes monitoring *Facebook* and *Twitter* feeds, joining *Facebook* groups created for a specific cause or geographical area, following hashtags and using keywording to find leads and sources. Bolledo (2020) also emphasized the need to fact-check information gathered using these methods, highlighting the importance of news values and the 5Ws and one H in reporting. Second, to address the barriers in online press briefings, journalists organized themselves to raise their unanswered questions in media group chats of government organizations (Ilagan, 2020). Third, the NUJP organized peer support networks critical for minimizing stress and trauma among journalists who reported about and during COVID-19. Finally, in an effort to prevent contracting and spreading the virus among co-workers, journalists are maintaining records of their activities and a list of sources whom they interacted for purposes of contact tracing (Camus, 2020). The new methods employed in health reporting, as creative responses to the constraints brought upon by COVID-19, partly illustrate how an emerging practice may turn into professional norm (Henderson and Hilton, 2018) in health reporting during pandemic.

### Double-edged sword

At the onset of COVID-19, journalism in the Philippines has struggled with ongoing technological changes that bring about double-edged consequences. On one hand, digital media has enabled journalists to help Filipinos make better sense of the pandemic – from reporting infections and deaths regularly to covering press conferences organized by agencies at the frontlines of COVID-19 response. Through *Facebook* live videos, *Zoom*, and other video conferencing applications, journalists are able to talk about their lived experiences in covering COVID-19. Various groups inside and outside of the Philippines have been hosting a series of webinars on how to cover the pandemic. Media groups in the Philippines meanwhile have also organized press briefings that tackle the state of news reporting in the country. In the forum titled ‘Intrepid Journalism in the Time of Corona’ organized by *This Side Up Manila*, two journalists discussed the state of news from the early stages of the pandemic to the declaration of enhanced community quarantine (ECQ). Early in the live video, they shared their frustrations about the consequences of COVID-19 on fieldwork and storytelling. According to the reporters, covering COVID-19 is different from reporting about natural disasters or conflict zones because they felt that there was no end in sight to the pandemic. As a result, they reminded themselves and their colleagues to find a balance and slow down as the pandemic may be prolonged and even put the lives of their families at risk. These webinars, which are in theory accessible to anyone in the world, also allow journalists to share their experiences with and learn from their counterparts in other countries. For instance, *Hivos* organized a webinar titled ‘Data Driven Reporting During Covid-19’ with journalists from the Philippines, Kenya, and Mexico to find out how they have been affected by and coping with the pandemic. The journalists said they have found collaboration or working with other journalists and members of the academe and civil society as key in reporting when fieldwork is not possible. Like the Philippines, too, Kenya and Mexico also experience barriers in accessing and reporting information while their governments too are also mandating policies that could restrict press freedom (Hivos, 2020).

On the other hand, digital media has complicated the work of journalists as they had to deal with the spread of health mis- and/or disinformation. To partly explain the diffusion of online fake news (e.g. mass testing and vaccines), we engage with Tandoc et al. (2018) who emphasized the characteristics of technology and the role of audiences. For instance, social media made it challenging for journalists to delineate information sources from each other, especially given the evolving science of COVID-19. Because science is evolving, journalists tend to rely heavily on expert opinion, without verifying the experts’ assumptions. Correcting mis- and/ or disinformation about the pandemic was likewise difficult because journalists had limited understanding of what counted as fake news among Filipinos. Another problem that journalists had to contend with while working during the pandemic is the recent ‘data breach’ that used *Facebook* profiles of real people (Robles et al., 2020). The rise of fake *Facebook* accounts is counterproductive not just to fight against health mis- and/or disinformation but also places the identities of journalists at risk. To a large extent, the proliferation of health mis- and/ or disinformation is inextricably connected to the social context not just of COVID-19 but also the Philippines. As Tandoc et al. (2018) pointed out, ‘fake news needs the nourishment of troubled times in order to take root. Social tumult and divisions facilitate our willingness to believe news that confirms our enmity toward another group’ (p. 149). While it created new issues, COVID-19 has also reinforced existing problems in the country and one of those is the shrinking space for free speech.

### Shutdowns, suspensions, and shrinking spaces

The pandemic is also laying bare pre-existing conditions hounding the Philippine press in a supposed democracy. For instance, the government passed ‘The Bayanihan to Heal as One Act’ (Republic Act No. 11469) to give the president emergency powers that would enable him to quickly respond to COVID-19. Human rights and media advocates criticized this law as it included a provision penalizing ‘fake news’, which can easily be used and abused by those in power to file complaints against individuals, including journalists (Freedom for Media, Freedom for All Network, 2020). Again this posed another challenge to journalists and the audience who both use social media as a means to get and share information. In similar vein, the passage of the ‘Anti-Terrorism Act of 2020’ (Republic Act No. 11479) received pushback for its broad provisions. Human rights groups also say that the law has essentially also criminalized intent, which could send a chilling effect especially among journalists who might be working on stories critical of the government.

On 5 May 2020, ABS-CBN, the country’s largest media network, went off-air after its broadcast franchise expired. The House of Representatives, which oversees the granting of franchises, refused ABS-CBN’s bid for a renewal, which ultimately led the media giant to close its broadcast operations and lay off thousands of employees. This development comes after the conviction of Rappler executive editor Maria Ressa and former researcher-writer Reynaldo Santos Jr for supposedly violating the Cybercrime Prevention Act of 2012 (Republic Act No. 10175). The shutdown is seen as the latest in a series of attacks and threats against news organizations deemed as critical of the current administration (Gutierrez, 2020; Pago, 2020). Community journalism is neither spared. At least half of some 60 community newspapers have suspended or ceased printing due to economic losses caused by the quarantine, according to estimates from the Philippine Press Institute, the national association of newspapers. The NUJP also raised economic difficulties confronting many freelance journalists, especially those who work on contract in broadcast, since the start of the lockdown. Suspension of operations means that contractual media workers would not be able to earn because work is not available. The halt in the production of news by ABS-CBN and various papers across the archipelago means that people, especially those in far-flung areas, have fewer sources of news at a time when getting information is most crucial. Again, these developments point to how pandemic reportage may be tied to political landscape in the country (Henderson and Hilton, 2018).

## Conclusion

COVID-19 is transforming the practice and business of journalism. On one hand, the pandemic and the ensuing quarantine restrictions have prompted news organizations and journalists to adapt and take advantage of digital media to continue gathering and presenting news. On the other hand, the pandemic has also exposed journalists and audiences alike to further mis- and/ or disinformation as well as to government’s new efforts to stamp out ‘fake news’. These developments run in parallel with threats to press freedom and journalist safety. In a pandemic, journalists are not mere observers or mere reporters as they also face the same risks everyone else is exposed to (CNN Philippines, 2020b). By laying out the current media environment in this essay, we hope to expand and deepen the conversation between and among journalists, policymakers, and healthcare professionals about public health reporting. In line with Larsson et al. (2003), we encourage further conversations between journalists and healthcare professionals to collectively identify gaps in health reporting and broaden understanding of ‘fake news’ and how it thrives in social media. Consistent with Tandoc et al. (2018), we also recommend that journalists and healthcare professionals listen to their audience to help understand what counts as health-related ‘fake news’ for them. Moreover, we invite policymakers to protect democratic spaces that enable journalists, healthcare professionals, and citizens alike to gather and share information related to COVID-19. At a time when disseminating reliable information and holding the powerful to account have never been more critical, we deem it necessary to understand where journalists are coming from to understand both the long-standing and emerging issues they have to grapple with in a pandemic.
